# Fungus-derived hydroxyl radicals kill hepatic cells by enhancing nuclear transglutaminase

**DOI:** 10.1038/s41598-017-04630-8

**Published:** 2017-07-06

**Authors:** Ronak Shrestha, Rajan Shrestha, Xian-Yang Qin, Ting-Fang Kuo, Yugo Oshima, Shun Iwatani, Ryutaro Teraoka, Keisuke Fujii, Mitsuko Hara, Mengqian Li, Azusa Takahashi-Nakaguchi, Hiroji Chibana, Jun Lu, Muyi Cai, Susumu Kajiwara, Soichi Kojima

**Affiliations:** 1 Micro-Signaling Regulation Technology Unit, RIKEN Center for Life Science Technologies, Wako, Saitama Japan; 2 School of Life Science and Technology, Tokyo Institute of Technology, Yokohama, Kanagawa Japan; 3 Condensed Molecular Materials Laboratory, RIKEN, Wako, Saitama Japan; 4 Medical Mycology Research Center, Chiba University, Chiba, Chiba Japan; 5 China National Research Institute of Food and Fermentation Industries, Beijing, China

## Abstract

We previously reported the importance of induced nuclear transglutaminase (TG) 2 activity, which results in hepatic cell death, in ethanol-induced liver injury. Here, we show that co-incubation of either human hepatic cells or mouse primary hepatocytes derived from wild-type but not TG2^−/−^ mice with pathogenic fungi *Candida albicans* and *C. glabrata*, but not baker’s yeast *Saccharomyces cerevisiae*, induced cell death in host cells by enhancing cellular, particularly nuclear, TG activity. Further pharmacological and genetic approaches demonstrated that this phenomenon was mediated partly by the production of reactive oxygen species (ROS) such as hydroxyl radicals, as detected by a fluorescent probe and electron spin resonance. A ROS scavenger, N-acetyl cysteine, blocked enhanced TG activity primarily in the nuclei and inhibited cell death. In contrast, deletion of *C. glabrata nox-1*, which encodes a ROS-generating enzyme, resulted in a strain that failed to induce the same phenomena. A similar induction of hepatic ROS and TG activities was observed in *C. albicans*-infected mice. An antioxidant corn peptide fraction inhibited these phenomena in hepatic cells. These results address the impact of ROS-generating pathogens in inducing nuclear TG2-related liver injuries, which provides novel therapeutic targets for preventing and curing alcoholic liver disease.

## Introduction

The liver acts as the first barrier to the spread of bacteria as well as fungi present in intestine. There is growing evidence on the important role of gut microbiota such as the bacteria-liver interaction in the pathogenesis of alcoholic steatohepatitis (ASH) and non-alcoholic steatohepatitis (NASH)^1–4^. However, the role of intestinal fungi still remains much unclear. Recently, sequencing of the fecal mycrobiome showed overgrowth of *Candida albicans* (*C. albicans*) in ASH patients^5^. Among the different *Candida* species, the opportunistic pathogens *C. albicans* and *C. glabrata* rank as the two most common species in the human digestive tract and are responsible for 65–75% of systemic candidiasis, which has a high morbidity and mortality rate^6, 7^. In immunocompromised cases, these fungi might invade gastrointestinal mucosa to reach the liver and cause severe fungal infections^8, 9^. Hepatic *Candida* infection is the most commonly recognized complication in patients with acute leukaemia and other haematological malignancies that prominently involve the liver^9^. A prospective study performed 25 years ago reported that fungal infection was present in 32% of patients with acute liver failure and that *Candida* species were the principle fungus present, although the underlying molecular mechanisms of these infections remain to be elucidated^10^.

Transglutaminase 2 (TG2, EC 2.3.2.13) is the most ubiquitously expressed Ca^2+^-dependent protein-crosslinking enzyme implicated in the regulation of cell growth, differentiation and apoptosis^11^. Previously, we addressed the role of induced cellular TG activity in hepatic cell death during the pathogenesis of both alcoholic and non-alcoholic steatohepatitis via crosslinking and inactivation of the general transcription factor Sp1, which resulted in the decreased expression of growth factor receptors essential to cell survival^12, 13^. Intracellular reactive oxygen species (ROS) have been reported to activate TG2 in different cell types^14–16^. Intriguingly, TG2 exhibits multiple additional functions in the regulation of cell growth and death depending upon the cell type and stimuli^17^. In dying cells, intracellular ROS enhances TG2 activation, which facilitates Bax translocation to the mitochondria. Thus, the release of cytochrome *c* and apoptosis-inducing factors from the mitochondria can induce both caspase-dependent and caspase-independent apoptotic cell death, respectively^18^.

Here, by investigating the cellular activity of TG2 in a human hepatic cell line (HC cells) and mouse primary hepatocytes following co-incubation with *Candida* species, we explored the hypothesis that these fungi might induce the nuclear activity of TG2 in hepatic cells. We show that ROS-producing fungi such as *C. albicans* and *C. glabrata* are associated with enhanced cellular activity, particularly nuclear TG activity, in hepatic cells, which led to apoptosis. A similar phenomenon was reproduced in the livers of mice injected with *Candida* species. We found that co-incubation of hepatic cells with opportunistic fungi, such as *C. albicans* and *C. glabrata*, but not edible yeasts, such as *Saccharomyces cerevisiae*, induces hepatic cell death by enhancing TG activity, at least in part through the production of ROS, such as hydroxyl radicals. An irreversible inhibitor of TG2, 6-diazo-5-oxo-norleucine tetrapeptide (ZDON)^19^, inhibited *C. albicans*-induced cell death as measured by caspase-3 activation. Further pharmacological and genetic approaches demonstrated that this phenomenon was mediated partly by intracellular ROS. A specific inhibitor of ROS, N-acetyl cysteine (NAC)^14^, inhibited the induction of cellular TG activity and cell death. Deletion of an *NADPH* oxidase gene (*NOX1*) in *C. glabrata*, which encodes a ROS-generating enzyme, failed to induce the same phenomena. These findings provide additional mechanistic insights into the exacerbation of liver (tissue) injury by pathogenic fungi and the importance of ROS removal for liver protection.

## Results

### Co-incubation of *C. albicans* or *C. glabrata* with HC cells increased cellular TG and caspase-3 activity levels in HC cells

**Figure 1:**
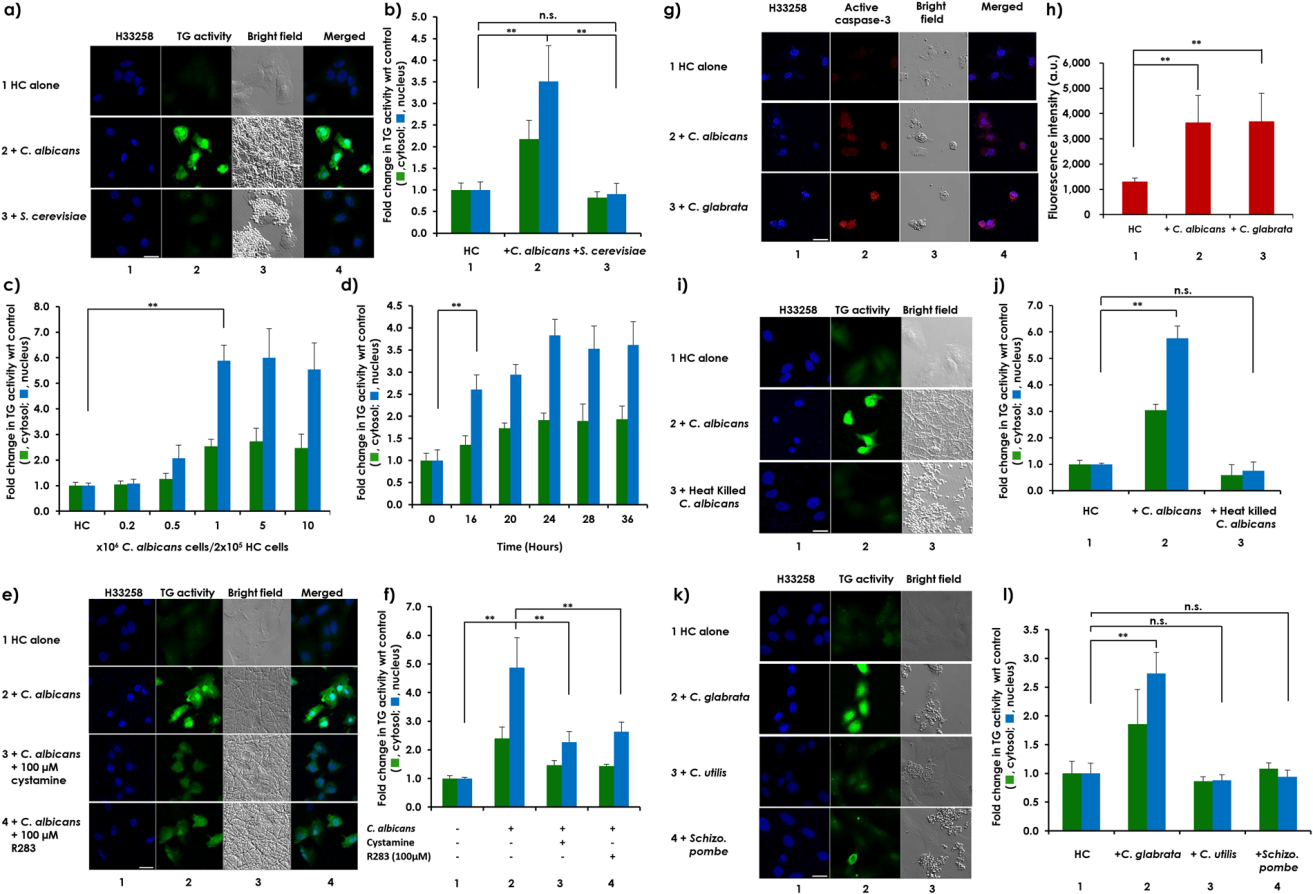
Cellular TG activity in HC cells increased, especially in the nucleus, upon co-incubation with pathogenic fungi. HC cells were seeded at 2 × 10^5^ cells per well in a 6-well plate and incubated overnight. After adding 5-BAPA, the cells were incubated (**a**) alone (row 1) or were co-incubated with 2.5 × 10^6^ *C. albicans* cells (row 2) or *S. cerevisiae* cells (row 3) for 24 hours; (**c**) with different doses of *C. albicans* cells for 24 hours; (**d**) with 5 × 10^6^ *C. albicans* cells for the indicated time; (**e**) with 5 × 10^6^ *C. albicans* cells in the absence (row 2) and presence of 100 µM of TG2 inhibitors, cystamine (row 3) or R283 (row 4) for 24 hours; (**g**) alone (row 1) or were co-incubated with either 5 × 10^6^ *C. albicans* cells (row 2) or the same number of *C. glabrata* cells (row 3) in an insert cup with a 0.4-µm pore size; (**i**) alone (row 1) or were co-incubated with living 5 × 10^6^ (row 2) or heat-killed 5 × 10^9^ (row 3) *C. albicans* cells for 24 hours; or (**k**) alone (row 1) or were co-incubated with living 5 × 10^6^ *C. glabrata* (row 2), *C. utilis* (row 3) and *Schizo. pombe* cells (row 4) for 24 hours. Scale bars = 20 µm. Representative images from at least 3 fields from 3 independent experiments are shown for (**a,e,I** and **k**) and from at least 3 fields from a single experiment for (**g**). Fluorescence intensities from TRITC in both the cytoplasm and nucleus of panels (b,c,d,f and j) were quantitated using ZEN 2011 software, and relative TG activity levels in both locations are presented in bar graphs, with the levels from HC cells incubated alone (panels b,c,f,h and j) or at time 0 (**d**) used as the controls (mean ± SD, n = 3). Fluorescence intensities from Alexa 488 signal from panel (**h**) were quantitated using ZEN 2011 software, and the mean ± SD are presented in bar graphs after subtraction of background intensities. Asterisks represent statistical significance.

Co-incubation of a hepatic cell line (HC) with *C. albicans*, but not with *S. cerevisiae*, enhanced the cellular incorporation of 5-(biotinamido)pentylamine (5-BAPA), a TG substrate (Fig. 1a and b, compare rows or columns 1 with 2 and 3, respectively). Maximum 2.5- and 6-fold increases in TG activity were observed in cytosolic and nuclear regions, respectively, in a dose- and time-dependent manner, reaching a plateau after the co-incubating of 2 × 10^5^ HC cells with 5 × 10^6^ *C. albicans* cells for 24 hours (Fig. 1c and d). Both cystamine (a broad TG inhibitor) and R283^20^ (a site-directed specific TG inhibitor) significantly inhibited *C. albicans*’ induction of cellular TG activity in HC cells (Fig. 1e and f, rows or columns 3 and 4, respectively), suggesting that more than 60% of the detected TG activity in HC cells was induced by *C. albicans*. TG2 mRNA levels were also enhanced in HC cells upon co-incubation with *C. albicans* for 8 hours (Fig. S1a). In EGFP-TG2-overexpressing HC cells, co-incubation with *C. albicans* for 24 hours caused a nuclear accumulation of the overexpressed TG2 (Fig. S1b and S1c). Although no significant decrease in the number of HC cells was observed after co-incubation for 24 hours, the cells became smaller in size. However, further co-incubation to 48 hours resulted in caspase-3 activation and cell death (Fig. 1g and h, compare rows and columns 1 with 2). In contrast, heat-killed *C. albicans* lost its capacity to increase TG activity in HC cells (Fig. 1i and j, compare rows or columns 1 with 3). Another pathogenic species, *C. glabrata*, but not the edible species *C. utilis* or the fission yeast *Schizosaccharomyces pombe*, showed a capacity to induce cellular TG activity similar to that of *C. albicans* (Fig. 1k and l, compare rows or columns 1 with 2, 3 and 4, and Fig. 1g and h, compare rows and columns 1 with 3). Next, pharmacological approaches were employed to determine whether inhibition of TG2 activation might affect fungus-induced hepatic cell death. An irreversible inhibitor of TG2, ZDON, significantly inhibited *C. albicans*-induced cell death as measured by caspase-3 activation in a human hepatocarcinoma functional liver cell-7 (FLC-7) cell line (Fig. S2). In addition, a nuclear TG2 inhibitor, phenosafranine, which inhibits nuclear localization of TG2 without affecting the transaminase activity itself^21^, also significantly inhibited *C. albicans*-induced caspase-3 activation in FLC-7 cells (Fig. S2), suggesting that nuclear TG2 activity is involved in *C. albicans*-induced hepatic cell death. Furthermore, the effect of *C. albicans* infection was compared between TG2 wild-type (TG2^+/+^) and knockout (TG2^−/−^) mice. Infection with *C. albicans* administrated via tail vein induced death of the animals in a dose-dependent manner **(**Fig. S3a **)**. Although both showed time-dependent decreases in body weight after infection with a non-lethal dose of 4 × 10^5^ *C. albicans*, a significantly attenuated body weight loss was observed in TG2^−/−^ mice compared to TG2^+/+^ mice **(**Fig. S3b and S3c **)**.

### Co-incubation with pathogenic *Candida* species increased levels of ROS in HC cells

**Figure 2:**
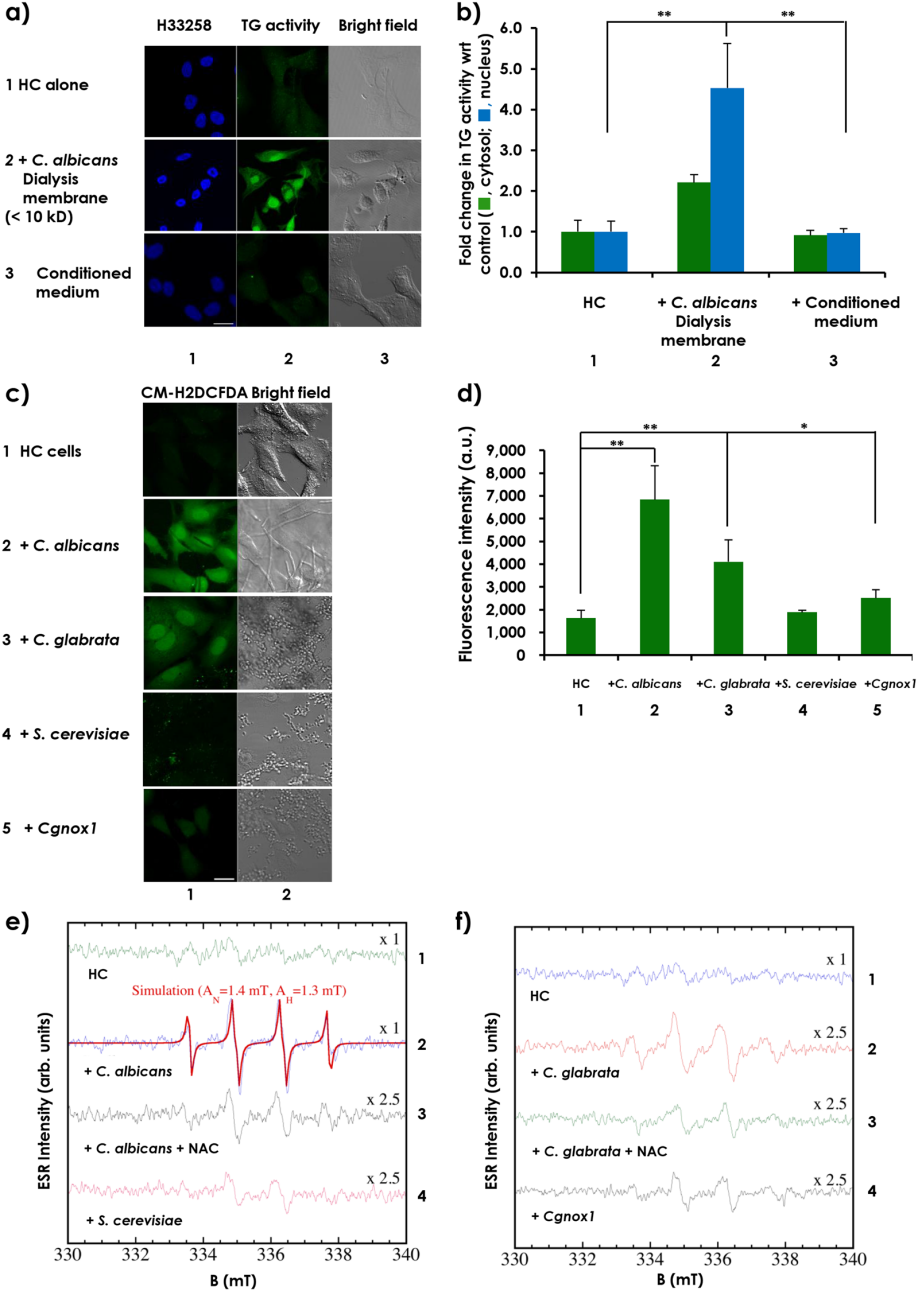
Levels of ROS in HC cell were increased following co-incubation with pathogenic *Candida* species. HC cells were seeded at 2 × 10^5^ cells per well on a 35-mm glass-based dish or round cover slips plated in 6-well plates and were incubated overnight. (**a**) After adding 5-BAPA, the cells were incubated alone (row 1) or were co-incubated with 5 × 10^6^ *C. albicans* cells grown in a transwell separated by a dialysis membrane (row 2) or with the conditioned medium prepared from *C. albicans* cultures following a 24-hour incubation (row 3). Representative images from at least 3 fields from 3 independent experiments are shown. (**b**) Fluorescence intensities of TRITC in both the cytoplasm and nucleus were quantitated using ZEN 2011 software, and the relative TG activity levels are presented in bar graphs with the levels from the HC cells incubated alone as the control (mean ± SD, n = 3). (**c**) Cells were incubated alone (row 1) or were co-incubated for 8 hours with the different fungal strains, including *C. albicans* (row 2), *C. glabrata* (row 3), *S. cerevisiae* (row 4), and the *CgNOX1* gene-targeted mutant of *C. glabrata* (row 5), with 5 µM CM-H2DCFDA. Scale bars = 20 µm. (**d**) Fluorescence intensities from reacted CM-H2DCFDA were quantitated using ZEN 2011 software and are represented in bar graphs (mean ± SD, n = 3). A 100-µL test mixture was prepared containing 40 µL DPTA, 10 µL BMPO and (**e**) 50 µL of culture medium from the HC cells (spectrum 1) or HC cells co-incubated with *C. albicans* in the absence (spectrum 2) or presence (spectrum 3) of NAC or *S. cerevisiae* (spectrum 4) cultivated for 8 hours or with (**f**) 50 µL of culture medium from HC cells (spectrum 1), or HC cells were co-incubated with *C. glabrata* in the absence (spectrum 2) or presence (spectrum 3) of NAC or *Cgnox1* (spectrum 4) for 8 hours. The mixture was transferred to a quartz flat cell and set inside the cavity of the ESR spectrometer and analyzed using the data analyzer connected to the ESR spectrometer.

Because induction of cellular TG activity required co-incubation with living pathogenic fungi, we wanted to estimate the molecular size of the mediator produced from fungi. To this end, HC cells were co-incubated with *C. albicans* plated in a dialysis membrane (cutoff <10 kDa). Increased cellular TG activity was observed in HC cells treated with the fungus in transwell plates (Fig. 2a and b, compare rows or columns 1 with 2) but not in HC cells treated with the fungus-conditioned media (Fig. 2a and b, compare rows or columns 1 with 3). This finding suggests that a certain soluble factor(s), which might be very unstable in nature, was secreted into the culture medium by *C. albicans* and caused increased cellular TG activity. Suspecting that unstable substances, such as ROS, might act as promising mediators, and ROS generation was measured using the fluorescent probe 5–6-chloromethyl-2′,7′-dichlorodihydrofluorescein diacetate (CM-H2DCFDA)^22^ (Fig. 2c and d). Significantly higher levels of ROS were produced by both *C. albicans* (row and column 2) and *C. glabrata* (row and column 3) than by HC cells alone (row and column 1) or by *S. cerevisiae* (row and column 4). The capacities of *C. albicans* and *C. glabrata* to produce higher levels of ROS may characterize them as pathogenic fungi. In *C. glabrata*, the only known gene (CAGL0K05863g) homologous to the genuine NADPH oxidase Yno1p/Aim14p of *S. cerevisiae* ^23^ was identified (and named *CgNOX1)* and was determined to be responsible for the generation of superoxide from oxygen. We prepared *Cgnox1*-disruption mutants and confirmed the decrease in ROS generation (row and column 5).

To determine the ROS species, electron spin resonance (ESR) analyses were performed, and high levels of hydroxyl radicals (˙OH) were identified in freshly harvested HC cells co-cultured with conditioned medium from *C. albicans* (Fig. 2e) and *C. glabrata* (Fig. 2f), but not in the medium of the *Cgnox1* mutants, relative to the HC cell-conditioned medium (Fig. 2e and f). The order of the ˙OH spectrum intensities was *C. albicans* > *C. glabrata* > *S. cerevisiae*, with an approximate ratio of 10:3:1, respectively (Fig. 2e and f). The ˙OH spectrum intensity of *Cgnox1* was also quite small (almost the same as that of *S. cerevisiae*). Treatment of HC cells and *C. albicans*/*C. glabrata* co-cultures with NAC also showed spectra similar to that of ˙OH but with different hyperfine parameters (A_H_ = 1.5 mT A_N_ = 1.4 mT). We speculate that this might be an extrinsic effect due to the interactions of NAC, ˙OH and 5-tert-butoxycarbonyl-5-methyl-1-pyrroline-N-oxide (BMPO). These results indicate that certain levels of exogenous ROS trigger the induction of nuclear TG in adjacent hepatic cells.

### *Candida* species-derived ROS-induced enhancement of cellular and, to a greater extent, nuclear TG activity in HC cells

**Figure 3:**
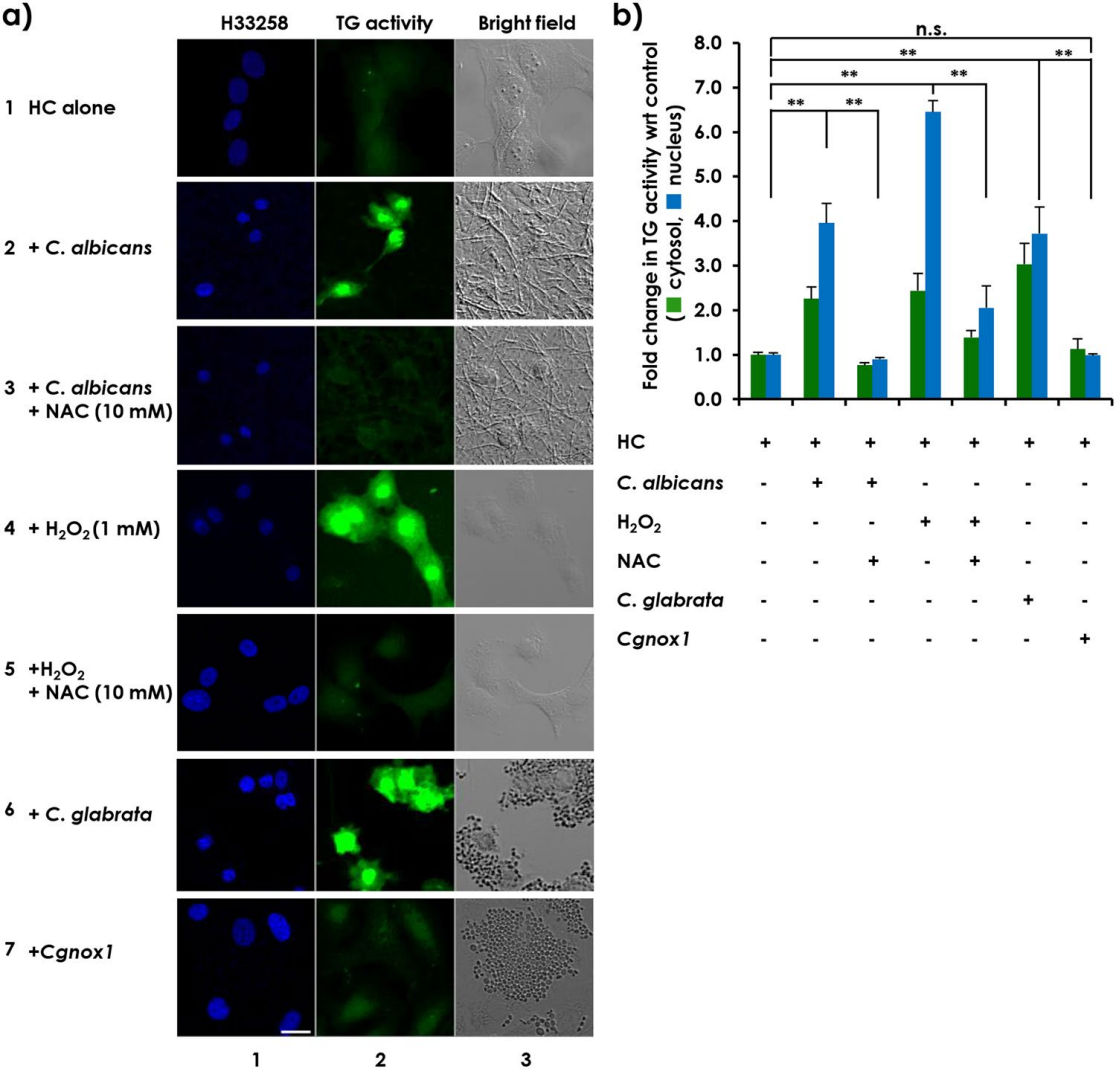
ROS produced by *C. albicans* induced cellular TG activity in HC cells. HC cells were seeded at 2 × 10^5^ cells per well in a 35-mm glass-based dish or on round cover slips plated in 6-well plates and incubated overnight as previously described. (**a**) After adding 5-BAPA, the HC cells were incubated alone (row 1) or were co-incubated for 24 hours with 5 × 10^6^ *C. albicans* cells in the absence (row 2) or presence (row 3) of 10 mM NAC, with 1 mM H_2_O_2_ in the absence (row 4) or presence (row 5) of 10 mM NAC, or with 5 × 10^6^ *C. glabrata* cells (row 6) or *Cgnox1*-mutant cells (row 7). The cells were fixed and stained with streptavidin-TRITC and H33258 dye. Both the blue fluorescence signals from H33258-stained nuclei (column 1) and the green fluorescence signals representing TG activity (column 2) were detected using a confocal microscope. Images taken under the bright field (column 3) are also shown. Scale bars = 20 µm. Representative images from at least 3 fields from 3 independent experiments are shown. (**b**) Fluorescence intensities from TRITC in both the cytoplasm and nucleus were quantitated using ZEN 2011 software, and the relative TG activity levels in both locations are represented in bar graphs, with the level from HC cells incubated alone used as the control (mean ± SD, n = 3).

To explore whether fungus-derived ROS might mediate induction of cellular TG activity in HC cells, cells were co-incubated with *C. albicans* in the presence and absence of NAC, an inhibitor of ROS^14^. Treatment of the co-cultures of *C. albicans* and HC cells with NAC completely blocked the enhanced cytosolic and nuclear TG activities in HC cells (Fig. 3a and b, compare rows or columns 2 with 3). These results suggest that ROS might mediate the enhancement of cellular and, to a greater extent nuclear, TG activity in HC cells. This hypothesis was verified by both gain- and loss-of-function experiments. First, to examine the capacity of ROS to induce increased TG activity, HC cells were treated with H_2_O_2_ in the presence or absence of NAC. Similar to HC cells co-incubated with *C. albicans*, externally added H_2_O_2_ mimicked an increase in cellular TG activity in HC cells, which was blocked by NAC (Fig. 3a and b, compare rows or columns 4 with 5). However, HC cells co-incubated with the *Cgnox1* mutant failed to increase TG activity (Fig. 3a and b, compare row or column 6 with 7). These results suggest that the ROS produced during co-incubation of HC cells with *C. albicans* and *C. glabrata* in the proximity of HC cells worked as a mediator(s) to increase cellular TG activity in HC cells.

**Figure 4:**
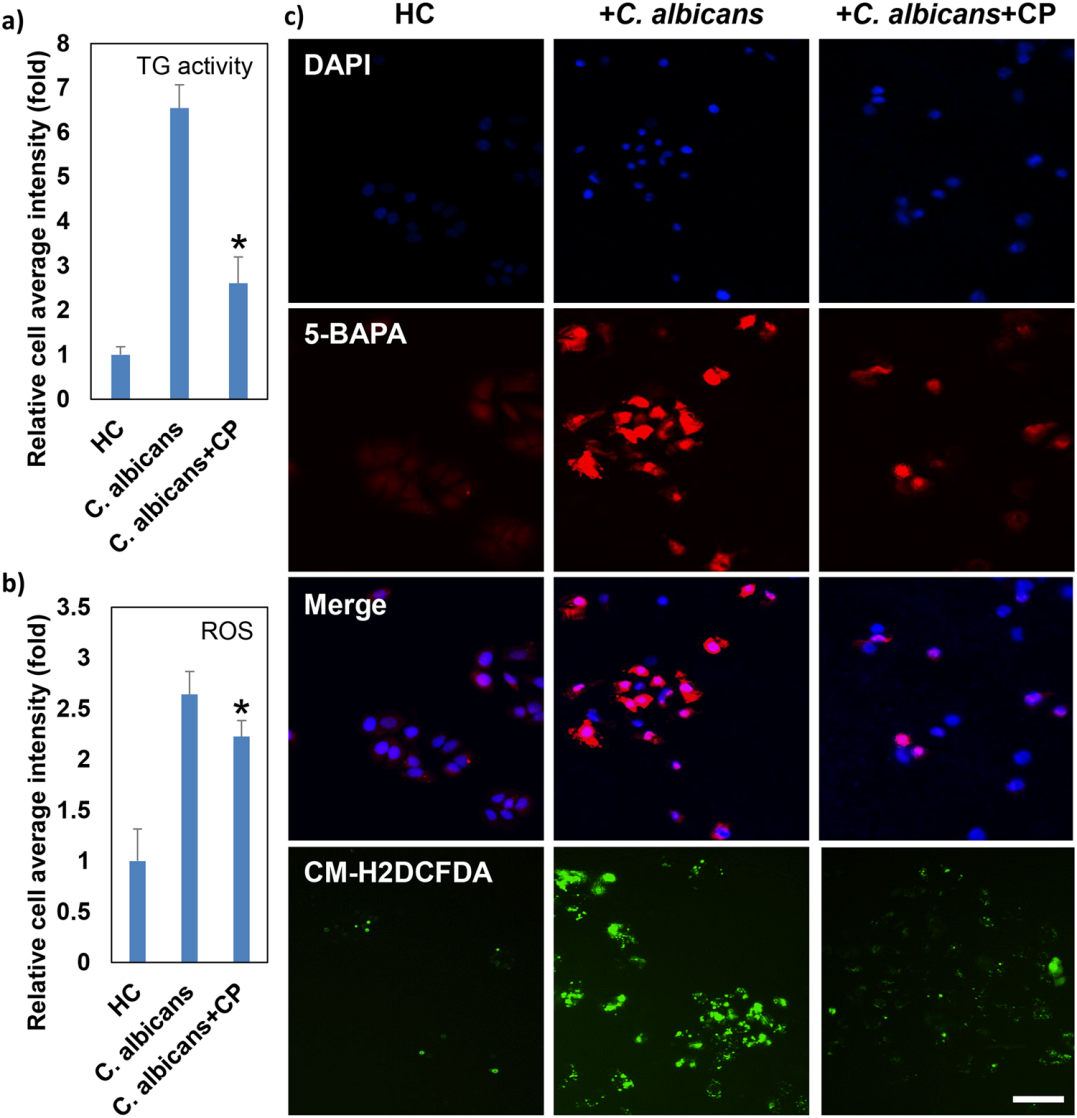
Antioxidant corn peptide fraction inhibits *C. albicans*-induced TG activity in HC cells. HC cells were seeded at 1 × 10^4^ cells per well in a 96-well plate and were incubated overnight as previously described. Cells were incubated alone (HC) or were co-incubated with 1 × 10^6^ *C. albicans* cells (*C. albicans*) in the presence of 100 μg/mL corn peptide fraction (*C. albicans* + CP). (**a**) TG activity in the cells was detected by 5-BAPA incorporation, as previously described. After 24 hours, cells were fixed and stained with streptavidin-TRITC and 4′,6-diamidino-2-phenylindole (DAPI) dye. Both the blue fluorescence signals from DAPI-stained nuclei and the red fluorescence signals representing TG activity were detected with the ImageXpress^MICRO^ High Content Screening System, and morphological analysis was performed using MetaXpress Image Analysis software. (**b**) After 24 hours, cells were incubated with 5 µM CM-H2DCFDA for ROS detection. Green fluorescence signals from CM-H2DCFDA representing ROS were observed with the ImageXpress^MICRO^ High Content Screening System (Molecular Devices, Sunnyvale, CA, USA), and morphological analysis was performed using MetaXpress Image Analysis software (Molecular Devices). The representative quantitative data are presented as the mean ± SD of at least three replicates from two independent experiments. (**c**) Representative fluorescence images from at least 4 fields from more than three samples of two independent experiments. Scale bar = 50 μm.

This possibility gave rise to another intriguing question of whether naturally produced antioxidants derived from food could be inhibitors of ROS production and applied to prevent and manage pathogenic fungal infection-induced TG activity^24^. Therefore, we screened an antioxidant corn peptide fraction (CP), which inhibited *C. albicans*-induced TG activity in HC cells (Fig. 4a and c) concomitantly with an inhibition of *C. albicans*-induced ROS generation in HC cells (Fig. 4b and c). Furthermore, these results also demonstrated that in combination with high content screening technology, the *in vitro* co-culture system of pathogenic fungi and hepatic cells presented in this study provided a unique and powerful tool for the discovery of nuclear TG inhibitors.

### *Candida* species-derived ROS-induced caspase-3 activity following increased cellular TG activity in HC cells and mouse primary hepatocytes

**Figure 5:**
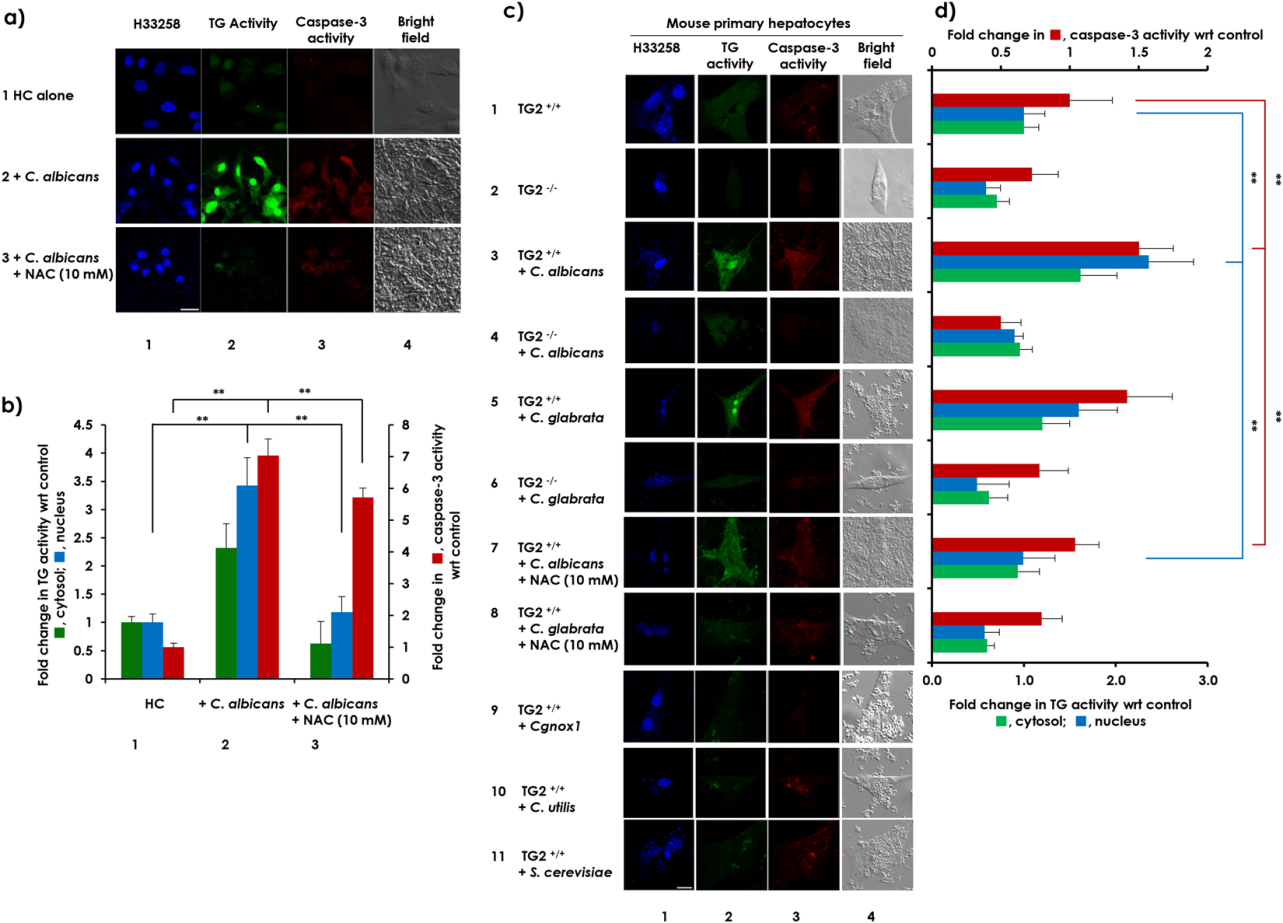
ROS derived from *C. albicans and C. glabrata* induced cell death in HC cells and mouse primary hepatocytes. (**a**) HC cells were seeded at 2 × 10^5^ cells per well in a 6-well plate and were incubated overnight and then treated with 5-BAPA and were incubated alone (row 1) or were co-incubated with 5 × 10^6^ *C. albicans* cells (row 2) in the presence of 10 mM NAC (row 3). (**c**) Primary hepatocytes isolated from livers of TG^−/−^ mice and their TG^+/+^ littermates were seeded at 5 × 10^5^ cells per well in a 6-well plate and incubated overnight. The next day, the cells were treated with 5-BAPA and incubated alone (rows 1 and 2) or were co-incubated with 5 × 10^6^ *C. albicans* cells (rows 3 and 4) in the presence of 10 mM NAC (row 7) or with the same number of *C. glabrata* cells (rows 5 and 6) in the presence of 10 mM NAC (row 8), *Cgnox1* (row 9), *C. utilis* (row 10), and *S. cerevisiae* (row 11). The blue fluorescence from H33258 dye (column 1) was observed along with the green fluorescence signals from TRITC staining 5-BAPA representing TG activity (column 2) and red fluorescence from Alexa 488 representing caspase-3 activity (column 3) using a confocal microscope. An image taken under bright field (column 4) is also shown. Scale bar = 20 µm. Representative images from at least 3 fields from a single experiment are shown. (**b** and **d**). Fluorescence intensities from TRITC in both the cytoplasm and nucleus along with fluorescence intensities from Alexa 488 were quantitated using ZEN 2011 software, and after subtraction of background intensities, the relative TG and caspase-3 activity levels are represented in bar graphs, with the levels from HC cells or TG2^+/+^ primary hepatocytes incubated alone as the control (mean ± SD, n = 3).

Next, to explore the role of ROS in caspase-3 activation, HC cells were incubated with pathogenic fungi. Enhanced cellular TG activity and caspase-3 activation were observed and were attenuated by NAC (Fig. 5a and b, compare row or column 2 with 3). Primary hepatocytes from TG2^+/+^ and TG^−/−^ mice were treated with different strains of fungi (Fig. 5c and d). Increased cellular TG and caspase-3 activity levels were observed following co-culturing *C. albicans* or *C. glabrata* with TG2^+/+^ hepatocytes (compare rows 1 with 3 and 5, respectively) but not with TG2^−/−^ hepatocytes (compare rows 2 with 4 and 6, respectively) or TG2^+/+^ hepatocytes treated with *Cgnox1*(row 9), *C. utilis* (row 10) or *S. cerevisiae* (row 11). The increased TG activity and caspase-3 activation were attenuated by NAC (compare rows 3 with 7 and 5 with 8), indicating that fungi-derived ROS increases cellular TG activity, especially TG2, and increases caspase-3 activation, leading to cell apoptosis.

### *In vivo C. albicans* infection-induced ROS production and TG activity in mouse livers

Finally, to evaluate whether injection of pathogenic fungi can also induce *in vivo* hepatic TG activity, mice were infected with *C. albicans* administered via the tail vein (Fig. S4a). Three days after injection, a significant decrease in body weight was observed in *C. albicans-*infected mice relative to the control mice (Fig. S4b). Histological and fluorescence staining of the liver sections showed that there were more inflammatory cell infiltrations and higher levels of ROS in livers infected with *C. albicans* (Fig. S4c and S4d). To evaluate *in vivo* TG activity, 30 minutes prior to euthanasia, mice were injected with approximately 100 μg/g of 5-BAPA by intraperitoneal injection. Mice were then euthanized, and sections of fresh-frozen tissue were stained with streptavidin-TRITC for immunofluorescence detection. As the results indicate, increased TG activity was observed in the periportal area in *C. albicans*-infected mouse livers, suggesting that the induction of TG activity due to pathogenic fungal infection might contribute to liver injury (Fig. S4e).

## Discussion

Although the roles of the gut microbiota and bacteria-liver interactions are fairly well understood in the context of ASH and NASH^1–4^, the role of intestinal fungi still remains unclear. Recently Yang *et al*. reported the induction of *C. albicans* in ASH patients^5^. Here, we demonstrated, for the first time, an association of ROS-producing fungi, such as *C. albicans* and *C. glabrata* with enhanced nuclear TG2 activity in hepatic cells leading to apoptosis, illustrating the impact of ROS-generating pathogens in inducing or exacerbating nuclear TG2-related liver injuries, which may provide a molecular mechanism of hepatic injury observed in ASH/NASH patients.

We found that *C. albicans* and *C. glabrata* produced high levels of ROS. This ability may be characterized as one of the pathogenic factors of *C. albicans* and *C. glabrata*. This result is consistent with the findings of Schröter *et al*.^25^, who found that *C. albicans* generates ROS and releases it extracellularly. Indeed, hydroxyl radicals were detected in the conditioned medium freshly isolated from those fungi. Reproducible enhancement of cellular TG activity by fungal ROS and exogenously added H_2_O_2_ were observed, while the ROS scavenger NAC blocked these effects. Furthermore, a mutation in *NOX1* in *C. glabrata*, which eliminated its capacity to produce ROS, abrogated the capacity of *C. glabrata* to enhance cellular TG activity. These results suggest that the ROS produced by *C. albicans* and *C. glabrata* in close proximity to HC cells acted as a principal mediator(s) in increasing cellular, especially nuclear, TG activity in the HC cells and, eventually, induced cell death (Fig. 5). A similar phenomenon was reproduced in *C. albicans-*infected mouse livers (Fig. S4). However, we cannot rule out the possibility that *C. albicans* and *C. glabrata* might also stimulate endogenous ROS production in HC cells. We are currently investigating the underlying molecular mechanisms of the production of high levels of ROS by *C. albicans* and *C. glabrata* and those for the ROS-induced enhancement of cellular TG activity. ROS can directly inactivate TG2^26^. Thus far, we have observed that ROS enhances both the gene expression and nuclear localization of TG2 in HC cells.

Increased intracellular ROS production has been reported to be the principal mediator for TG2 activation in human umbilical vein endothelial cells (HUVEC) lines treated with C-peptide^16^, in Swiss 3T3 cell lines treated with lysophosphatidic acid (LPA) and transforming growth factor beta (TGF-β)^14^, and in NIH3T3 cell lines treated with arachidonic acid^15^. In these studies, endogenously produced ROS enhanced cellular TG activity, although the biological relevance of this enhancement was not addressed. In addition, it has been reported that *C. albicans* produces ROS under certain conditions^25^. Combining these previous findings and the results of the current study suggests that host cell (tissue) injury by *Candida* via the ROS-mediated induction of nuclear TG2 might be a general phenomenon. Indeed, we observed a similar induction of nuclear TG2 in HEK293T cells, a human embryonic kidney cell line, upon co-incubation with *C. albicans* (data not shown). Concomitant bacterial infection with fungal infection has been previously reported^10, 27^. We are currently investigating the effect of ROS-generating bacteria on the regulation of cellular TG activity in hepatic cells.

**Figure 6:**
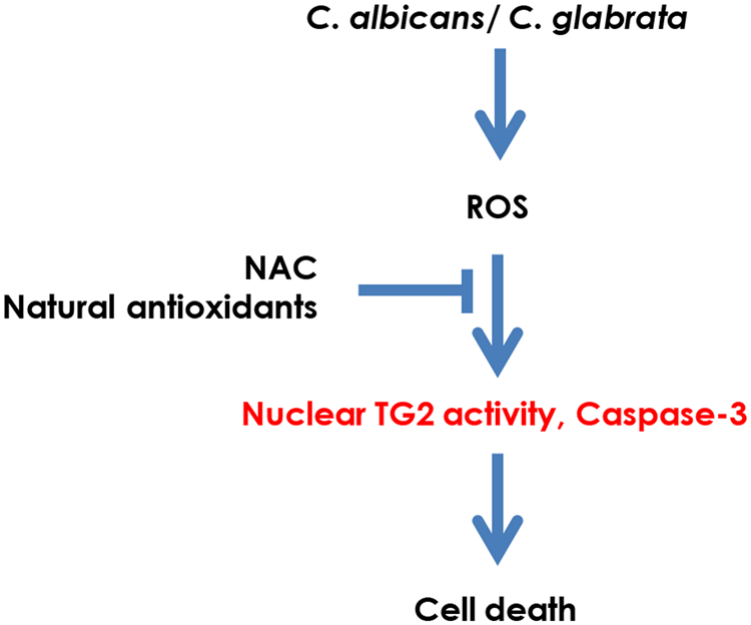
Schematic diagram showing the mechanism of *C. albicans*- and *C. glabrata-*induced cell death in HC cells. Both fungi produce ROS that induces cellular TG activity leading to HC cell death. NAC and natural antioxidants are effective in preventing this cell death by blocking the action of ROS.

In conclusion, in this study, we demonstrate that the pathogenic fungi *C. albicans* and *C. glabrata* increase cellular TG activity levels in hepatic cells. This increase eventually leads to cell death due to the release of ROS, which acts as a principal mediator (Fig. 6). This observation suggests that the production of high levels of ROS and the ROS-mediated induction of nuclear TG2 might be a basic feature of pathogenic fungi. ROS plays a vital role in host defense and has been involved in various pathological conditions, including cardiovascular disease^28^, neurodegenerative diseases such as Alzheimer’s disease^29^ and Parkinson’s disease^30^, diabetes^16^ and renal cell carcinoma^31^. TG2 has also been associated with these diseases^32–36^. In such situations, suitable inhibitors of exogenous ROS may serve as promising therapies for liver injury in hepatitis patients by inhibiting the enhancement of cellular TG activity.

## Methods

### Ethics Statement

The *in vivo* infection experiments were performed in accordance with protocols approved by the RIKEN Institutional Animal Use and Care Administrative Advisory Committee (H28-2-002(3)) and adhered to the guidelines of the Institutional Regulation for Animal Experiments and Fundamental Guidelines for Proper Conduct of Animal Experiments and Related Activities in Academic Research Institutions under the jurisdiction of the Ministry of Education, Culture, Sports, Science and Technology, Japan.

### Cell culture

HC cells from a human hepatic cell line were purchased from Cell Systems (Kirkland, WA, USA) and were grown in CS-C complete medium. FLC-7 cells were kindly supplied by Dr. Matsuura (The Jikei University School of Medicine, Tokyo, Japan)^37^. The cells were cultured at 37 °C in a humidified incubator with 5% CO_2_. For the experiments, cells were plated and cultured overnight in 96-well plates or 6-well plates bearing round coverslips. The next day, the cells were incubated for 4 hours in Dulbecco’s modified Eagle’s medium (DMEM) supplemented with 0.2% fetal bovine serum (FBS) for serum starvation (Mediatech, Herndon, VA, USA). These cells were co-incubated with fungi in the presence of 5-BAPA (Thermo Scientific, Rockford, IL, USA) in serum-free DMEM.

### Isolation of primary hepatocytes

Primary hepatocytes were isolated from the livers of male TG2^−/−^ mice and their TG2^+/+ ^^38^ littermates by collagenase digestion method as described previously^39^; the cells were then cultured in DMEM containing 10% FBS.

### Fungal strains and culture conditions

Each fungal strain (Supplemental Table S1) was grown on yeast peptone dextrose (YPD, 1% yeast extract, 2% peptone, 2% dextrose and 2% agar) agar medium. For co-incubation with HC cells and *in vivo* infection, the fungi were pre-cultured in liquid YPD medium with shaking at 160 rpm for 8 to 16 hours at 30 °C. The pre-cultures were washed twice with phosphate-buffered saline (PBS) and adjusted to the desired cell density in plates by measuring the optical density at 600 nm (OD600) using an Ultrospec 2000 spectrophotometer (Pharmacia Biotech, Piscataway, NJ, USA). For the preparation of heat-killed *C. albicans* cells, 5 × 10^9^ cells/mL from an overnight culture were washed twice with PBS and heated in DMEM at 70 °C for 20 minutes. Heat-killing of the cells was confirmed based on the loss of growth following the incubation of heated cells on YPD agar plates for 48 hours at 30 °C. Preparation of *NOX1*-disrupted *C. glabrata* mutants is detailed in the Supplemental Information.

### Determination of *in vitro* TG activity

HC cells were seeded on round coverslips plated in a 6-well plate (2 × 10^5^ cells) and incubated overnight at 37 °C with 5% CO_2_. The next day, the cells were directly or indirectly co-incubated for 24 hours with fungi using a transwell with a 0.4-μm pore size membrane or a dialysis membrane (cutoff < 10 kDa), creating a barrier between the HC cells and the fungi. The cellular activity of TG2 was measured based on the incorporation of 0.2 mM 5-BAPA into the cells incubated in the presence of 0.1 mM aminoguanidine with and without 100 µM cystamine or R283, which are TG2 inhibitors^20^. Some samples were also incubated with 50 μM ZDON (Zedira, Darmstadt, Germany)^19^ or 10 μM phenosafranine (Sigma-Aldrich Co., St. Louis, MO, USA)^21^ as TG2 inhibitors, 10 mM NAC (Sigma-Aldrich Co.) as an ROS inhibitor^14^, or 1 mM H_2_O_2_ as a positive control for producing ROS. Cells were then fixed with a 10% formaldehyde solution, permeabilized, blocked and immunostained with streptavidin-tetramethylrhodamine isothiocyanate (TRITC) (Jackson ImmunoResearch Laboratories, West Grove, PA, USA) and cleaved caspase-3 (Cell Signaling Technology, Danvers, MA, USA). The TG activity was then detected as a fluorescence signal from TRITC and analyzed with an LSM 700 laser scanning confocal microscope (Carl Zeiss, Inc., Germany) using ImageXpress^MICRO^ High Content Screening System (Molecular Devices, Sunnyvale, CA, USA). The morphological analysis was performed using MetaXpress Image Analysis software (Molecular Devices). Anti-rabbit Alexa 488 was used to detect cleaved caspase-3 along with Hoechst 33258 dye.

### Determination of *in vitro* ROS production

HC cells were seeded onto a 35-mm glass-based dish (2 × 10^5^ cells) and incubated at 37 °C with 5% CO_2_ overnight. The next day, cells were co-incubated with different strains of fungi (5 × 10^6^ cells) for 8 hours. ROS production was analyzed based on the incorporation of CM-H2DCFDA (final concentration, 5 µM) (Life Technologies, Eugene, OR, USA) for 15 minutes at 37 °C, and cells were immediately monitored for their fluorescein isothiocyanate (FITC) fluorescence signals using confocal microscopy or an ImageXpress^MICRO^ High Content Screening System (Molecular Devices). The morphological analysis was performed using MetaXpress Image Analysis software (Molecular Devices).

### Identification of ROS by ESR spectroscopy

Semi-quantitative measurements of the generated ROS were performed using a conventional spin trapping technique with ESR spectroscopy. Detailed methods are provided in the Supplemental Information. HC cells were seeded onto a 6-well plate (2 × 10^5^ cells) and incubated at 37 °C with 5% CO_2_ overnight. Cells were co-incubated with the indicated fungi (5 × 10^6^ cells) for 8 hours. Culture media were diluted in 100 mM phosphate buffer (pH 7.4) containing 25 µM diethylenetriaminepentaacetic acid (DPTA) (Sigma-Aldrich Co.) and 25 mM BMPO (Enzo Life Sciences, Farmingdale, NY, USA)^40^. The mixtures were then transferred to a quartz flat cell and set inside the cavity of the ESR spectrometer. Then, ESR measurements of the spin adducts were performed under the following conditions: magnetic field, 336.7 ± 10 mT; microwave frequency, 9.424 GHz; microwave power, 10.0 mW; sweep time, 0.5 minutes; time constant, 0.03 sec; and modulation field 1.0 × 0.1 mT (100 kHz). All ESR spectra were taken with 10 accumulation times. The generated ROS were evaluated using the data analyzer connected to the ESR spectrometer. Representative images obtained from a single experiment are shown.

### *In vivo* fungal infection

Six-week-old male C57BL6/J mice, and 8-week-old female and male TG2^+/+^ and male TG2^−/−^ mice^12^ were housed under constant temperature (22 °C ± 1 °C), with free access to food and water. To establish *in vivo* infection^41^, mice were injected via their lateral tail vein with approximately 4 × 10^5^, 4 × 10^6^, or 4 × 10^7^ cells/mouse of *C. albicans*. Mice were euthanized on the third or fourth day of infection after being anesthetized with isoflurane gas.

### Determination of *in vivo* ROS production

Frozen liver tissue sections were washed twice with PBS and stained with 5 μM CM-H2DCFDA and incubated for 30 minutes at 37 °C. Subsequently, the tissue was washed twice with PBS and mounting medium, and cover slips were placed on the slides. The FITC fluorescence signals were detected using a Zeiss LSM 700 laser scanning confocal microscope.

### Determination of *in viv*o TG activity

*In vivo* TG activity was assessed as previously reported^42, 43^. Briefly, 30 minutes prior to euthanasia, mice were injected with approximately 100 μg/g of 5-BAPA by intraperitoneal injection. Mice were then euthanized, and liver tissues were covered in optimal cutting temperature compound (OCT) and snap-frozen in liquid nitrogen. Sections (7 μm in thickness) were cut using a Leica sliding microtome (Leica Microsystems, Nussloch, Germany) and fixed in 4% paraformaldehyde. For histology, the sections were stained with Myer’s hematoxylin solution and 1% Eosin Y solution (Muto Pure Chemicals, Tokyo, Japan). For 5-BAPA staining, the sections were permeabilized with PBS/0.3% Triton X-100 and blocked with 5% goat serum in PBS at room temperature for 30 minutes. Thereafter, the sections were treated with streptavidin-TRITC (1:500 dilution, Jackson ImmunoResearch, West Grove, PA, USA) and Hoechst 33258 dye (1:5000 dilution, Wako Industries, Osaka, Japan) in blocking buffer at room temperature for 45 minutes. Immunofluorescence signals were detected using a Zeiss LSM 700 laser scanning confocal microscope.

### Statistical analysis

Quantitative data are shown as the mean ± SD (n = 3–5). Statistical analyses were performed using GraphPad Prism version 6.0 for Windows (GraphPad Software, San Diego, CA, USA). A *p-value < 0.05 and a **p-value < 0.01 were considered statistically significant.

## Electronic supplementary material

Supplementary methods, table and figures
