# Supplementary material for: Fungus-derived hydroxyl radicals kill hepatic cells by enhancing nuclear transglutaminase
Source: Sci Rep. 2017 Jul 6;7:4746. doi: 10.1038/s41598-017-04630-8 (PMC5500562; doi:10.1038/s41598-017-04630-8)

Supporting Information

Fungus-derived hydroxyl radicals kill hepatic cells by enhancing nuclear transglutaminase

Ronak Shrestha[1,2], Rajan Shrestha[1], Xian-Yang Qin[1], Ting-Fang Kuo[1], Yugo Oshima[3], Shun Iwatani[2], Ryutaro Teraoka[1], Keisuke Fujii[2], Mitsuko Hara[1], Mengqian Li[1], Azusa Takahashi-Nakaguchi[4], Hiroji Chibana[4], Jun Lu[5], Muyi Cai[5], Susumu Kajiwara[2] and Soichi Kojima[1]

[1] Micro-Signaling Regulation Technology Unit, RIKEN Center for Life Science Technologies, Wako, Saitama, Japan

[2] School of Life Science and Technology, Tokyo Institute of Technology, Yokohama, Kanagawa, Japan

[3] Condensed Molecular Materials Laboratory, RIKEN, Wako, Saitama, Japan

[4] Medical Mycology Research Center, Chiba University, Chiba, Chiba, Japan

[5] China National Research Institute of Food and Fermentation Industries, Beijing, China

Supplementary methods

Deletion of the *C. glabrata NADPH oxidase* (*Cg NOX1*) gene

A deletion mutant of the *C. glabrata NOX1* gene (CAGL0K05863g) was constructed from the parental strain KUE100 as previously described [1]. The target gene *NOX1* was replaced by a DNA cassette containing the *HIS3* gene via homologous recombination. The DNA cassette was prepared via PCR using the primers p11890F (5´-TGGTCTTTTGACTGCAGAAAAGCTTACGTATTATTTAGGTAATATTA GATGGTTAAGGCCGCTGATCACG-3´) and p11890R (5'-TTATCAGATAATTTAAAAT GACTGGATCATAAGGTAAGAAATATATAATTGAATTACATCGTGAGGCTGG-3'). The pHIS906 plasmid containing *HIS3* was used as a template for PCR, and transformation was performed as previously described [2]. To verify the integration of the DNA cassette into the ORFs in the transformants, colony PCR was carried out using the forward primers p22F (5'-CAGCTTTATCTCAGAAAACCAG-3') and p11890Kcheck (5´-AATCCCCTCTCACCGAATGG-3'). PCR products of 400 bp indicated that the DNA cassette had been integrated into the correct site in the genome. No PCR products were produced from the genomic DNA of KUE100, the parental strain, using the primer pairs. To verify the deletion of the *NOX1* gene from the genome in the transformants, colony PCR was carried out using p11890orf (5'-GGTGAAATAAGAAGACCAGC-3') and p11890TR (5'-CGCCCATCTGATAACAAAG-3'), which anneal to the ORF of *NOX1.* The 492-bp PCR product was produced when KUE100 was used as a template, but no product was formed with the transformants.

Preparation of corn peptide fraction

Spray-dried powder of corn protein (600g, Zhucheng Xingmao Corn Developing Co. , Ltd, Weifang, Shandong, China) was suspended in 6 L distilled water, then heated to 90 °C by water bath, added 10 g NaOH and stirred fully for 3 hours. The suspension was centrifuged at 3000 rpm for 15 minutes (DL-5200B, Shanghai Anting Scientific Instrument Factory, Shanghai, China). The insoluble protein sediment was re-suspended in 6 L distilled water and hydrolyzed with neutral protease from *Bacillus subtilis* (enzyme: substrate = 1: 50; Novozyme, Copenhagen, Denmark) and flavourzyme from *Aspergillus oryzae* (enzyme: substrate = 1: 100; Novozyme) at 50 °C for 4 hours. After hydrolysis, the enzymes were inactivated by heating at 100 °C for 30 minutes. The resulting hydrolysates were centrifuged at 3000 rpm for 15 minutes to remove the precipitate. Then, the supernatant was added 2% (w/v) activated carbon, stirred at 55 °C for 1 hour, and treated with filtering and enriched (RE5200, Shanghai Yarong Biochemistry Instrument Factory, Shanghai, China) to 30% (w/w). Finally, the dry powder of the oligopeptide fraction was produced by spray drying (Beijing Laiheng Lab-Equipments, Beijing, China).

Identification of ROS using ESR spectroscopy

Four split ESR line spectra with an intensity ratio of 1:2:2:1 were observed in the conditioned medium from *C. albicans* but not in that from HC alone (Fig. 2e). The origin of the split ESR lines comes from the hyperfine interactions of the nuclear spins and depends on the number of nuclear spins in the nitrone spin trap BMPO and the trapped ROS. If hydroxyl radicals (•OH) are trapped in BMPO, the total number of nuclear spins are I=3/2, and this splits the single ESR line into 2I+1 = 4 lines. The number of the splitting lines and the intensity ratio and the hyperfine parameters (A_N = 1.4 mT, A_H = 1.3 mT) suggests that the origin of the ESR was coming from the spin adducts BMPO/˙OH. Therefore, the ROS was identified as a

hydroxyl radical ($^{\cdot}OH$). The intensities of the $^{\cdot}OH$ spectra were quite different for the different species of fungi. *C. albicans* showed the highest intensity. *C. glabrata* showed a spectrum for $^{\cdot}OH$ at a 2.5-fold higher sensitivity setting (Fig. 2f), suggesting the amount of $^{\cdot}OH$ released by *C. glabrata* is relatively lower than that released by *C. albicans*.

Supplementary figures

Fig S1. ***C. albicans*** **enhanced both TG2 mRNA expression and nuclear translocation of overexpressed TG2 in HC cells.** HC cells were seeded at 2 × 10^5 cells per well in a 6-well plate and incubated overnight as previously described. **(a)** Cells were incubated alone (sample 1) or co-incubated either with 5 × 10^6 *C. albicans* cells in an insert cup with a 0.4-µm pore size (sample 2). After 8 hours, RNA was isolated from cell cultures using an RNeasy kit (Qiagen, Valencia, CA, USA). The purity of the isolated RNA was evaluated using a NanoDrop spectrophotometer (NanoDrop products, Wilmington, DE, USA). PrimeScript RT Master Mix Kit (TaKaRa Bio, Otsu, Japan) was used to synthesized cDNA, and PCR reactions were performed using a LightCycler® 96 (Roche Diagnostics) with SsoAdvancedTM SYBR® Green Supermix (Bio-Rad Laboratories, Hercules, CA, USA). Relative amounts of TG2 mRNA were plotted after normalization against the GAPDH mRNA levels in the same sample. **(b)** Cells were transiently transfected with 0.8 µg of *pEGFP-C1* or TG2-*pEGFP-C1*. Twenty-four hours after transfection, the cells were co-incubated with 5 × 10^6 *C. albicans* cells for another 24 hours. The cells were then fixed, stained with H33258 dye and observed with a confocal microscope. The blue fluorescence from the H33258 dye (columns 1 and 5) along with the green fluorescence of EGFP (columns 2 and 6) were monitored. Images taken under bright field (columns 3 and 7) and merged images are also shown (columns 4 and 8). Scale bars = 20 µm. Representative images from at least 3 fields from a single experiment are presented. **(c)** Numbers of the cells overexpressing EGFP or EGFP-TG2 in the nuclei of cells in a 320 µm^2 area were counted, and the percentage of cells overexpressing EGFP or EGFP-TG2 relative to the total number of cells was calculated and plotted.

Fig S2. TG2 inhibitor represses *C. albicans*-induced hepatic cell death. FLC-7 cells were seeded at 1×10^4 cells per well in a 96-well plate and incubated overnight as previously described. Cells were incubated alone or co-incubated with 5×10^7 *C. albicans* cells in the absence and presence of 50 μM ZDON (a synthetic specific inhibitor to TG2[3]) or 10 μM phenosafranin (an inhibitor to block nuclear localization of TG2[4]) for 24 hours. Cell death was determined by fluorescence intensity with ImageXpress^{MICRO} High Content Screening System (Molecular Devices, Sunnyvale, CA, USA) and morphological analysis was performed using MetaXpress Image Analysis software (Molecular Devices). **(a)** Representative fluorescence images from three biological samples of two independent experiments. Scale bar = 20 μm. DAPI: blue; cleaved caspase-3 (clCasp3): red. **(b)** The representative quantitative data of nuclear cleaved caspase-3 intensity were presented as mean ± SD from three biological samples of two independent experiments.

Fig S3. Effect of *C. albicans* infection on the body weight of TG2$^{+/+}$ and TG2$^{-/-}$ mice.

(a) Survival rates of C57BL6/J mice 1 or 2 days after injection with PBS (n=2) or *C. albicans* at doses of 4×10^5 cells/mouse (n=3), 4×10^6 cells/mouse (n=3) or 4×10^7 cells/mouse (n=2) by intraperitoneal injection. **(b)** Representative photos of TG2$^{+/+}$ and TG2$^{-/-}$ mice. **(c)** Changes in body weight in female TG2$^{+/+}$ mice 1 or 2 days after injection with PBS (n=2, blue line) or male TG2$^{+/+}$ (n=3, red line) and TG2$^{-/-}$ mice (n=3, green line) 1 or 2 days after injection with *C. albicans* at a dose of 4×10^5 cells/mouse.

Fig S4. Effect of *C. albicans* infection on TG2 activity in mouse livers. **(a)** Schematic overview of in vivo fungal experimental procedures (n=3). **(b)** Three days after injection, significant decrease in body weight was observed in *C. albicans* infected mice comparing with that of the control mice. Histological **(c)** and fluorescence staining with 5µM CM-H2DCFDA **(d)** of the liver sections showed that there were more inflammatory cell infiltration and higher levels of ROS in the liver infected with *C. albicans*. **(e)** Thirty minutes prior to sacrifice, mice were injected with approximately 100 µg/g of 5-BAPA by intraperitoneal injection. Mice were then sacrificed and sections of fresh-frozen liver tissues were stained with streptavidin-TRITC for immunofluorescence signals detection, which showed an increased TG activity in *C. albicans* infected mouse livers, especially in periportal area.

ST1. Different species of fungi used in the current study

Species	Strain	Genotype	References
C. albicans	SC5314	wild type	[5]
S. cerevisiae	IFO10150	wild type	[6]
C. glabrata	CBS138	wild type	[1]
C. utilis	IFO0988	wild type	[7]
Schizo. pombe	IAM04863	wild type	JCM†
Cg NOX1	CAGL0K05863g	*Cgnox1*Δ	This study

† Japan Collection of Microorganisms

References

1. Ueno, K. *et al.* Intestinal resident yeast Candida glabrata requires Cyb2p-mediated lactate assimilation to adapt in mouse intestine. *PLoS One* **6**, e24759 (2011).
2. Ueno, K. *et al.* Development of a highly efficient gene targeting system induced by transient repression of YKU80 expression in Candida glabrata. *Eukaryot Cell* **6**, 1239-1247 (2007).
3. McConoughey, S. J. *et al.* Inhibition of transglutaminase 2 mitigates transcriptional dysregulation in models of Huntington disease. *EMBO Mol Med* **2**, 349-370 (2010).
4. Furutani, Y., Toguchi, M., Shrestha, R. & Kojima, S. Phenosafranin inhibits nuclear localization of transglutaminase 2 without affecting its transamidase activity. *Amino Acids* (2016).
5. Fonzi, W. A. & Irwin, M. Y. Isogenic strain construction and gene mapping in Candida albicans. *Genetics* **134**, 717-728 (1993).
6. Kajiwara, S. *et al.* Polyunsaturated fatty acid biosynthesis in Saccharomyces cerevisiae: expression of ethanol tolerance and the FAD2 gene from Arabidopsis thaliana. *Appl Environ Microbiol* **62**, 4309-4313 (1996).
7. Tomita, Y., Ikeo, K., Tamakawa, H., Gojobori, T. & Ikushima, S. Genome and transcriptome analysis of the food-yeast Candida utilis. *PLoS One* **7**, e37226 (2012).

Fig. S1 *C. albicans* enhanced both TG2 mRNA expression and nuclear translocation of overexpressed TG2 in HC cells.

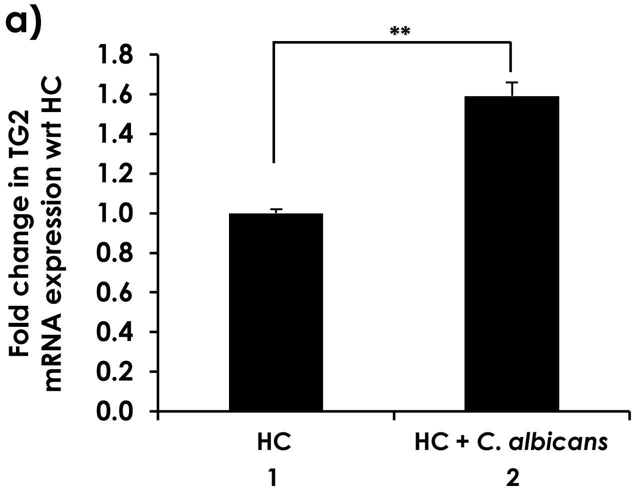

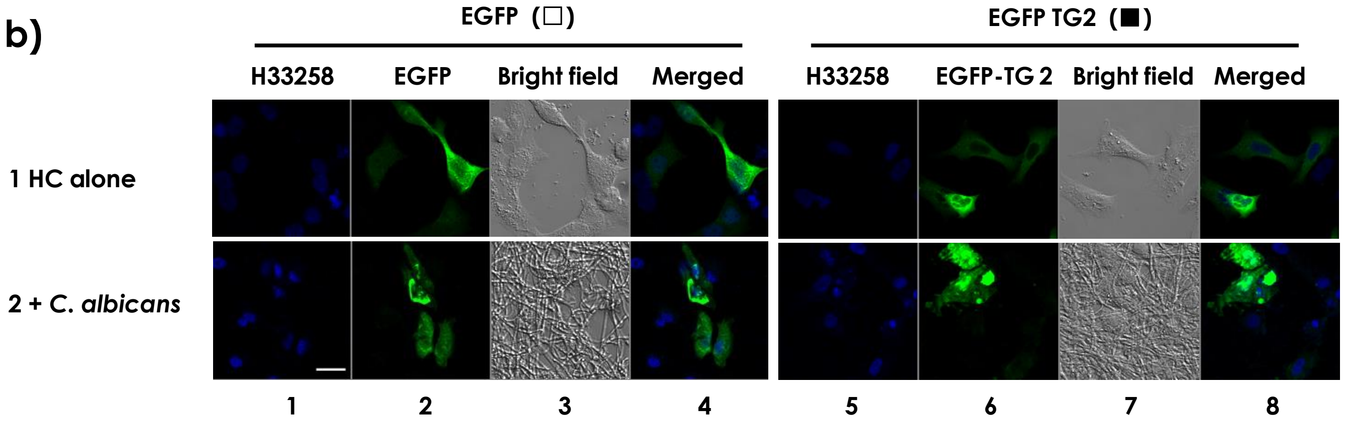

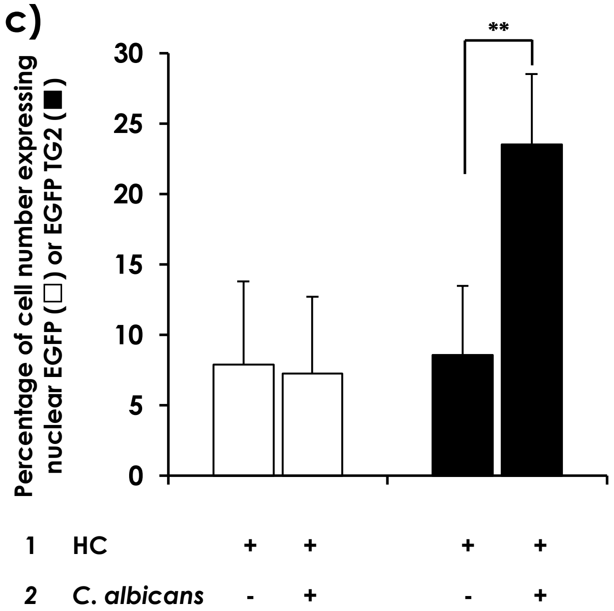

Fig. S2 TG2 inhibitor represses *C. albicans*-induced hepatic cell death

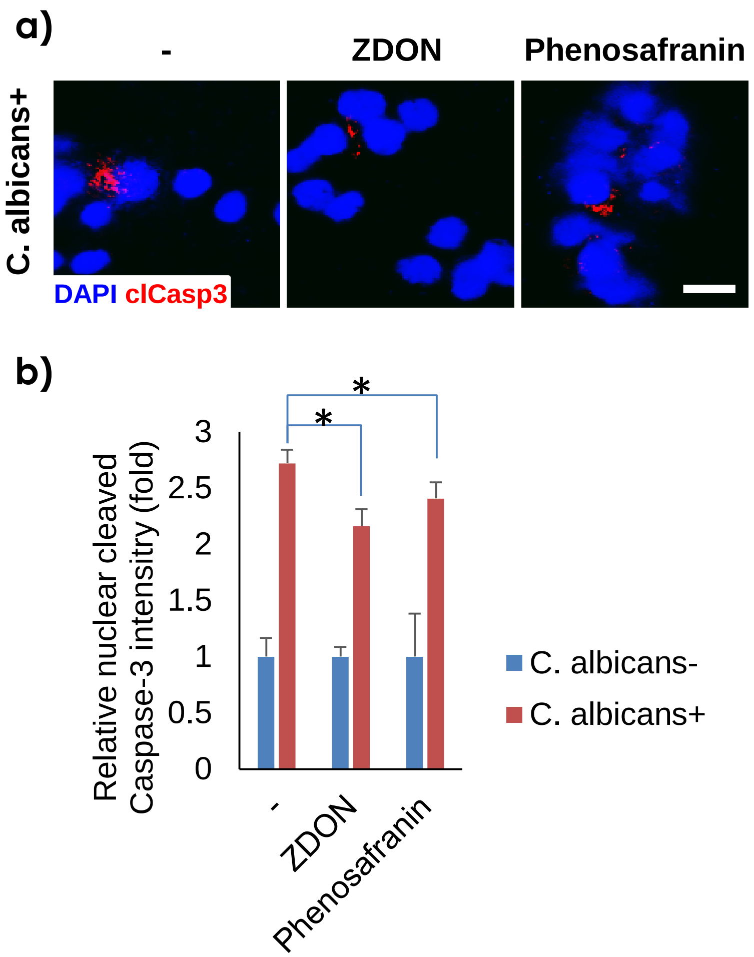

Fig. S3 Effect of *C. albicans* infection on the body weight of TG2$^{+/+}$ and TG2$^{-/-}$ mice.

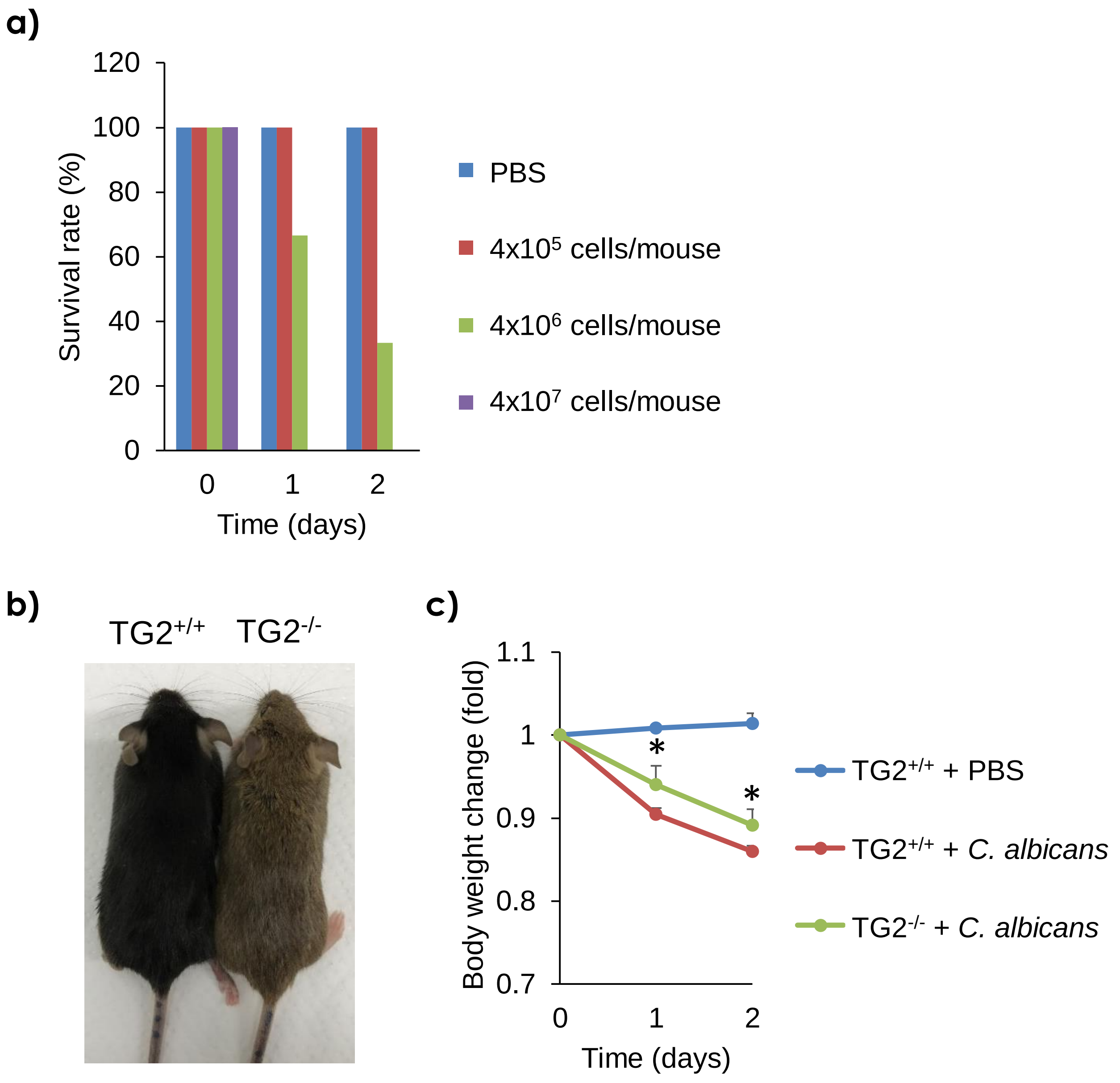

Fig. S4 Effect of *C. albicans* infection on TG2 activity in mouse livers.

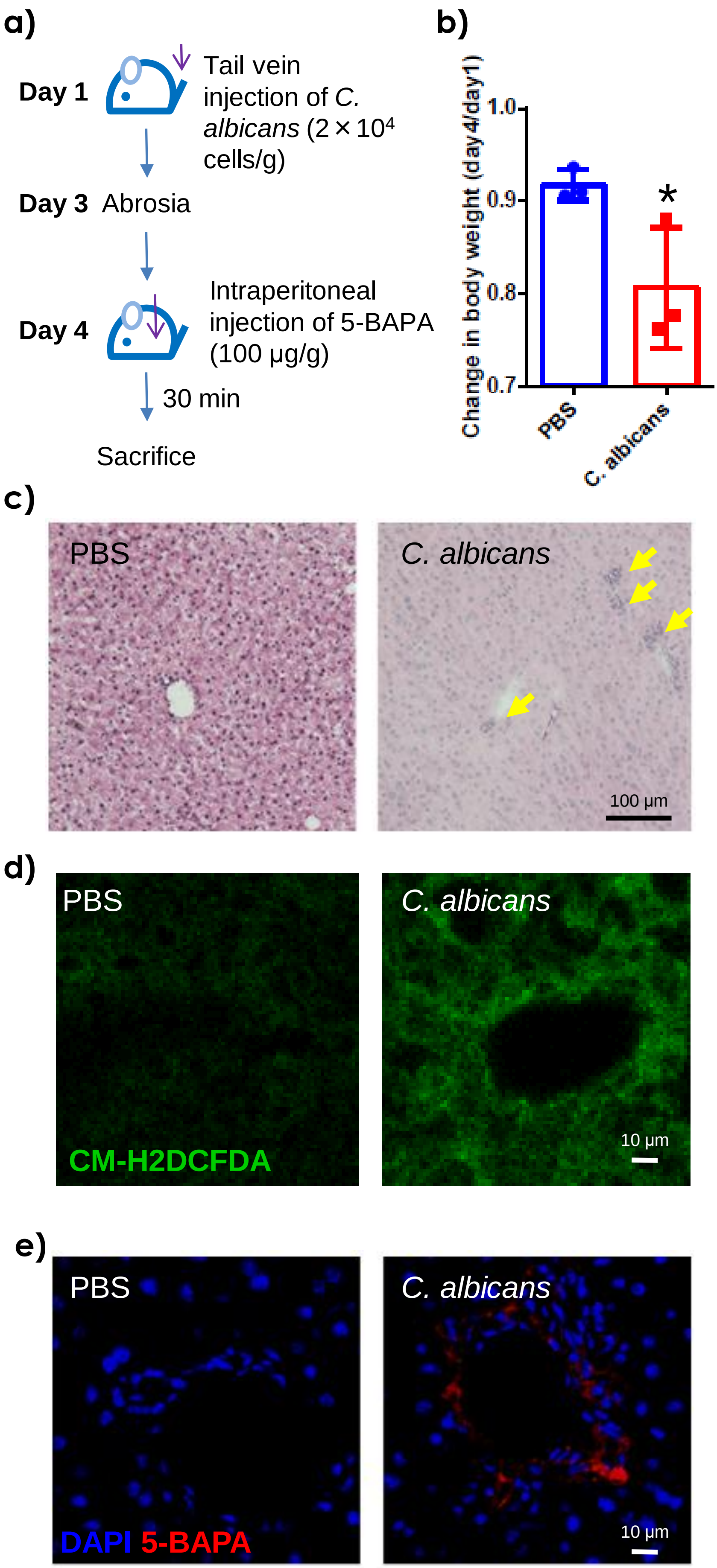

Supplement: Supplementary file 1 — Supplementary methods, table and figures [file 41598_2017_4630_MOESM1_ESM.pdf]
